# Post-ischemic stroke rehabilitation is associated with a higher risk of fractures in older women: A population-based cohort study

**DOI:** 10.1371/journal.pone.0175825

**Published:** 2017-04-17

**Authors:** Huei Kai Huang, Shu Man Lin, Clement Shih Hsien Yang, Chung Chao Liang, Hung Yu Cheng

**Affiliations:** 1 Department of Family Medicine, Buddhist Tzu Chi General Hospital, Hualien, Taiwan; 2 Department of Physical Medicine and Rehabilitation, Buddhist Tzu Chi General Hospital, Hualien, Taiwan; Medizinische Universitat Innsbruck, AUSTRIA

## Abstract

**Background:**

Rehabilitation can improve physical activity after stroke. However, patients may be more prone to falls and fractures because of balance and gait deficits. Few reports have studied the relationship between rehabilitation and subsequent fractures after ischemic stroke.

**Objective:**

To investigate whether post-stroke rehabilitation affects fracture risk.

**Methods:**

We conducted a population-based retrospective cohort study based on the Taiwan National Health Insurance Research Database. Patients with a newly diagnosed ischemic stroke between 2000 and 2012 were included. After propensity score matching, a total of 8,384 patients were enrolled. Half of the patients (4,192) received post-stroke rehabilitation within 1 month; the other half did not receive any post-stroke rehabilitation. Cox proportional hazards regression model was used to calculate hazard ratios (HRs) for fractures among patients with and without rehabilitation within 1 year after ischemic stroke. Patients were further stratified by sex and age (20–64 and ≥65 years).

**Results:**

Patients receiving post-stroke rehabilitation had a higher incidence of fracture (6.2 per 100 person-years) than those who did not (4.1 per 100 person-years) after adjustment for sociodemographic and coexisting medical conditions [HR = 1.53, 95% confidence interval (CI) = 1.25–1.87, p < 0.001]. The analyses performed after stratifying for sex and age showed that only older women undergoing rehabilitation had a significantly higher risk of fracture (HR = 1.62, 95% CI = 1.21–2.17, p = 0.001).

**Conclusion:**

Rehabilitation after ischemic stroke is associated with an increased fracture risk in older women.

## Introduction

Ischemic stroke is a devastating disease, resulting in disability in nearly half of the patients at hospital discharge [1]. It is also a major risk factor for hip fracture regardless of age or sex. It has been reported that stroke is associated with a two-fold increase in the risk of hip or femur fracture [2]. Compared with men, women with stroke have a higher fracture risk [3]. Ramnemark et al. found that more than 80% of all fractures after stroke were caused by falls and most frequently involved the femoral neck [4, 5]. Impaired balance may make it difficult to maintain erect upright posture while walking. Inability to maintain balance impairs functional performance and results in a higher frequency of falls [6]. Short- and long-term morbidity of fractures include pain, limitation of function, decreased health-related quality of life, and increased mortality [7]. Many studies have reported that hip [7–12] and spine fractures [7, 13–16] increase mortality in the general population.

Although rehabilitation enhances physical activity after stroke, it may be accompanied by falls due to balance and gait deficits [17]. Falls are common complications experienced during post-stroke rehabilitation [18, 19]. A prospective study estimated an incidence of 159 falls per 10,000 patient-days in an inpatient stroke rehabilitation setting, potentially subjecting patients to the serious complications of inactivity mentioned above. Acute fractures accounts for 4% of patients’ falling accidents during their rehabilitation stay [20]. To the best of our knowledge, there is no report on the long-term relationship between rehabilitation and fractures after ischemic stroke. We conducted a population-based cohort study to investigate the association between rehabilitation and fracture after ischemic stroke in Taiwan.

## Materials and methods

### Study design and data sources

The cohort data set was obtained from Taiwan’s National Health Insurance Research Database. In Taiwan, up to 99% of the population is covered by National Health Insurance (NHI), and 97% of hospital and clinics are contracted with NHI [21, 22]. For research purposes, the National Health Research Institute (NHRI) established the Longitudinal Health Insurance Database by randomly sampling a representative cohort of one million patients from the registry of all NHI beneficiaries from the year 2000 [23, 24]. There are no statistically significant differences in age, sex or health care costs between the sample cohort and all beneficiaries. We used this database to retrieve information about patient characteristics and medical records by linking ambulatory and inpatient care claims and the registry of beneficiaries. Because the data set used in this study comprised de-identified secondary data released to the public for research purposes, this study was granted exempt from review by the Institutional Review Board of the Tzu Chi Medical Center (REC No:IRB104-131C).

### Study population

Because ischemic stroke accounts for more than 80% of total strokes [25] and the claims-based stroke severity index (described below) is only applicable to ischemic stroke patients, our study population was defined as patients with ischemic stroke. To identify patients in whom new-onset ischemic stroke was diagnosed between 2000 and 2012, we used the primary discharge diagnosis of ischemic stroke (International Classification of Diseases, 9th Revision, Clinical Modification [ICD-9-CM] codes 433 and 434). The date of ischemic stroke diagnosis was recorded as the index date and the concurrent hospitalization as the index hospitalization. Rehabilitation recipients were defined as patients receiving either in-patient or out-patient post-stroke rehabilitation, according to NHI medical records. The rehabilitation aimed at functional improvement of hemiplegic limbs, muscle strengthening, posture, ambulation, and balance training.

**Fig 1:**
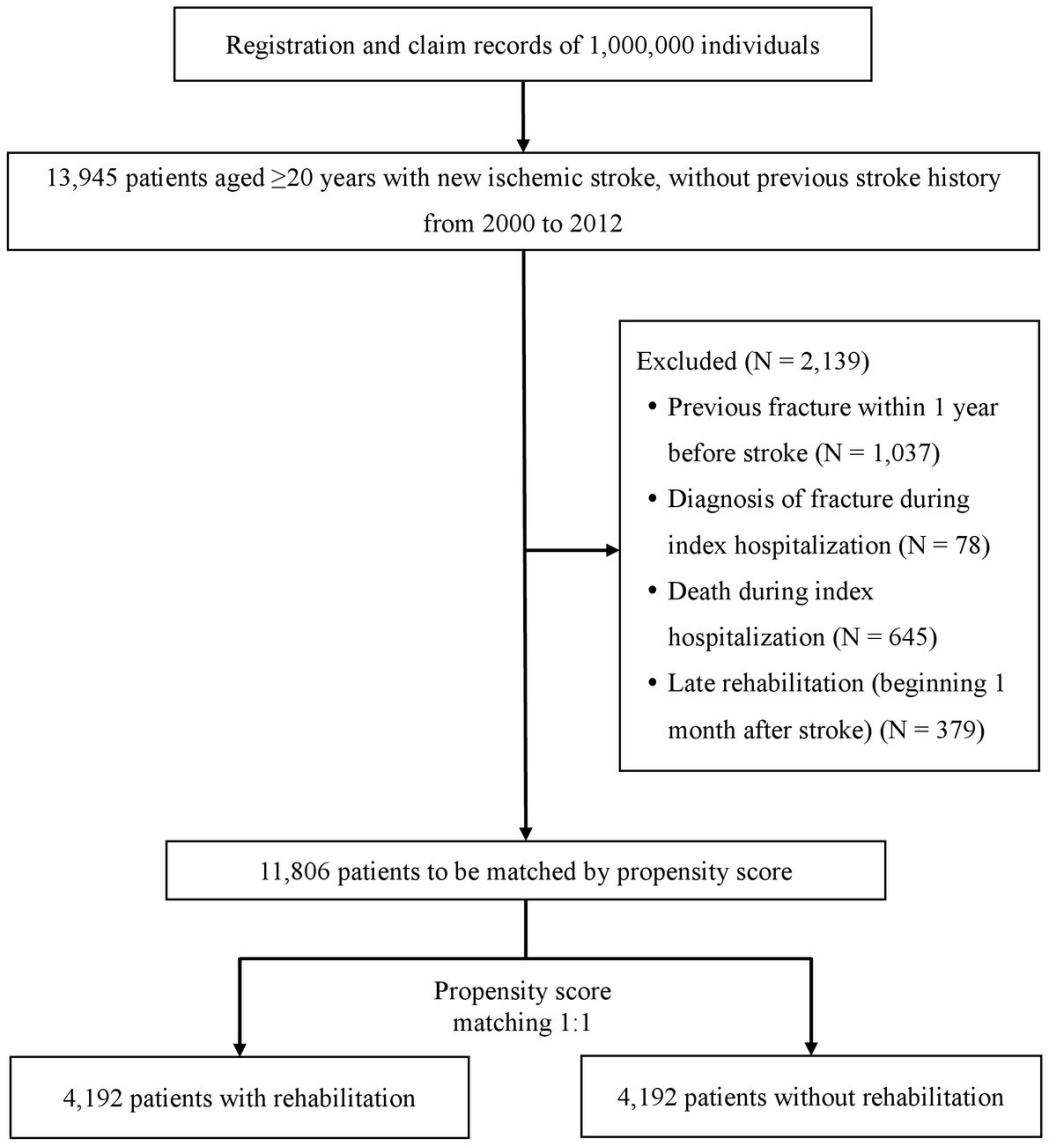
Flow diagram of selection of study subjects.

To evaluate an association between rehabilitation and fractures, patients were divided into two groups. The rehabilitation group included subjects who started post-stroke rehabilitation within 1 month after stroke, and the non-rehabilitation group comprised subjects who did not receive any post-stroke rehabilitation within 1 year after stroke. Exclusion criteria were: 1) age ≤20 years, 2) previous history of ischemic or hemorrhagic stroke, 3) fracture within 1 year before stroke, 4) diagnosis of fracture during index hospitalization, 5) death during index hospitalization, or 6) rehabilitation initiated 2 to 12 months after stroke (Fig 1).

### Primary outcome

ICD-9-CM codes from 800.X to 829.X were used to identify subjects with a new diagnosis of fracture within 1 year after ischemic stroke. All study participants were followed from the index date until a new diagnosis of fracture, withdrawal from the Longitudinal Health Insurance Database, or until 1 year after ischemic stroke. The ICD-9-CM codes for fractures were validated in previous studies and the accuracy could be acceptable [26, 27].

### Covariates

We identified comorbidities according to ICD-9-CM and procedure codes. A preexisting comorbidity was any disease diagnosed during at least one hospital admission or two outpatient visits in the 1 year preceding the index admission. Based on preexisting conditions identified from each patient’s medical records, Charlson comorbidity index scores were calculated [28]. Subjects’ demographic and clinical characteristics including age, sex, and comorbidities during index hospital admission were retrieved. Proxy indicators of stroke severity during the index hospitalization included Stroke Severity Index (SSI) score, diagnosis codes of hemiplegia, paraplegia, or aphasia; operation and procedure codes; mechanical ventilation; and intensive care unit admission [29]. The SSI score developed by Sung et al. was calculated to assess neurologic deficit severity of ischemic stroke [30]. The following seven features were used to calculate the SSI score: airway suctioning, bacterial sensitivity test, general ward stay, ICU stay, nasogastric intubation, osmotherapy, and urinary catheterization. Multiple linear regression model was used to calculate the SSI score based on the coefficients of these seven features reported by Sung et al. Previous studies revealed that the claims-based SSI correlated well with the National Institutes of Health Stroke Scale and the consequent functional outcomes after stroke [30, 31].

Socioeconomic data included the premium paid for NHI, which is set nationally according to income and is therefore a proxy for income. The premium cost was stratified in 4 levels: New Taiwan Dollars ≥40,000; 20,000–39,999; 1–19,999; and fixed. The fixed category included those designated by NHI as dependents, such as the unemployed, students, children, and elderly persons with no salary [32]. Subjects were also stratified by where they lived based on National Health Insurance Research Database information, identifying 7 levels of urbanization [33, 34], with level 1 indicating the most urbanized area. To simplify analysis, levels 5 through 7 were combined in a single group and counted as level 5 [32].

### Statistical analysis

To minimize selection bias, propensity score matching was performed to balance covariates and baseline differences, including age, sex, baseline comorbidities, socioeconomic factors, and stroke severity proxies (Table 1). We matched (without replacement) patients who had rehabilitation with those who did not. The nearest-neighbor algorithm was applied to construct matched pairs.

**Table 1:** Baseline characteristics and comorbidity of patients with and without post-stroke rehabilitation after propensity score matching.

	Rehabilitation (n = 4192)	Non-rehabilitation (n = 4192)	p value
**Demographic factors**			
Age (y), mean ± SD	68.0 ± 12.4	67.9 ± 12.4	0.774
Male, n (%)	2,533 (60.4%)	2,521 (60.1%)	0.789
**Comorbidities**			
Charlson Comorbidity Index	12.7 ± 1.85	2.72 ± 1.82	0.938
Hypertension	3,311 (79.0%)	3,310 (79.0%)	0.979
Diabetes mellitus	1,768 (42.2%)	1,744 (41.6%)	0.595
Osteoporosis	169 (4.0%)	171 (4.1%)	0.912
COPD	772 (18.4%)	736 (17.6%)	0.306
Congestive heart failure	386 (9.2%)	379 (9.0%)	0.791
Chronic kidney disease	371 (8.9%)	361 (8.6%)	0.699
Malignancy	256 (6.1%)	245 (5.8%)	0.612
Parkinsonism	128 (3.1%)	123 (2.9%)	0.749
Epilepsy	60 (1.4%)	64 (1.5%)	0.717
Dementia	208 (5.0%)	204 (4.9%)	0.840
Depression	179 (4.3%)	174 (4.2%)	0.786
**Socioeconomic factors**			
Insurance premium			0.186
Fixed	1,204 (28.7%)	1,249 (29.8%)	
1–19,999	1,658 (39.6%)	1,604 (38.3%)	
20,000–39,999	1,046 (25.0%)	1,015 (24.2%)	
≥40,000	284 (6.8%)	324 (7.7%)	
Urbanization level			0.029
1 (Most urbanized)	1,067 (25.5%)	1,073 (25.6%)	
2	1,119 (26.7%)	1,172 (28.0%)	
3	795 (19.0%)	722 (17.2%)	
4	709 (16.9%)	657 (15.7%)	
5 (Least urbanized)	502 (12.0%)	568 (13.6%)	
**Stroke severity proxies**			
SSI score	6.07 ± 3.88	6.11 ± 4.36	0.599
ICU	534 (12.7%)	544 (13.0%)	0.744
Mechanical ventilation	157 (3.8%)	156 (3.7%)	0.954
Aphasia	52 (1.2%)	62 (1.5%)	0.346
Hemiplegia or paraplegia	500 (11.9%)	551 (13.1%)	0.093
Neurosurgery	22 (0.5%)	21 (0.5%)	0.878

Continuous data expressed as mean ± standard deviation and categorical data expressed as number (%)

Abbreviations: COPD = chronic obstructive pulmonary disease, SSI = Stroke severity index, ICU = Intensive Care Unit

A χ2 test for categorical data and a t test for continuous variables were conducted to analyze differences between the rehabilitation and non-rehabilitation groups. Fracture-free rates were estimated with Kaplan–Meier curves, and the difference between survival curves was compared with the log-rank test.

Three different Cox proportional hazard regression models, including univariate, multivariate, and stratified Cox proportional hazard regressions, were conducted to calculate hazard ratios (HRs) and 95% confidence intervals (CIs) for fracture risk associated with post-stroke rehabilitation. We corrected for immortal time for all calculated incidence rates and HRs [35].

We used the same method of propensity score matching to perform analysis after stratifying for sex and age (20–64 and ≥65 years) on a total of 11,806 enrolled patients for assessing the association between rehabilitation and fracture risk. Database management and statistical analyses were performed using Stata version 13 (Stata Corporation, College Station, Texas, USA). A two-sided probability value of <0.05 was considered significant.

## Results

### Demographic characteristics of subjects

A total of 13945 adult patients with new-onset ischemic stroke were identified from the national cohort. After excluding patients not meeting study criteria and matching by propensity score, 8384 patients with ischemic stroke without recent or comorbid fracture were evaluated, of whom half (4192 subjects) received post-stroke rehabilitation (Fig 1).

Aside from a slight difference in the level of urbanization, there were no significant differences between the groups in terms of baseline characteristics, comorbidities, or stroke severity after propensity score matching (Table 1).

### Risk of fractures after stroke

**Fig 2:**
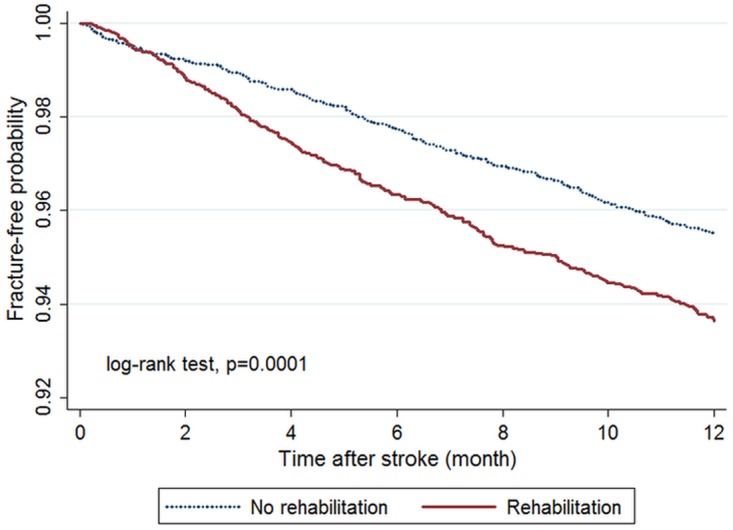
Kaplan–Meier curves showing estimated fracture-free proportions of patients with and without post-stroke rehabilitation.

The Kaplan–Meier model showed a higher cumulative incidence of fracture in the rehabilitation than in the non-rehabilitation group (6.2 vs 4.1 per 100 person-years; log-rank test, p = 0.0001) (Fig 2), with the difference appearing at about 2 months after stroke. Rehabilitation was associated with a higher risk of fracture during follow-up (crude HR = 1.53, 95% CI = 1.25–1.88, p < 0.001) (Table 2). This effect remained on multivariate Cox regression even after adjusting for all baseline characteristics listed in Table 1 (adjusted HR [aHR] = 1.53, 95% CI = 1.25–1.87, p < 0.001) and on stratified Cox regression by stratifying propensity-score matched groups (aHR = 1.52, 95% CI = 1.23–1.88, p < 0.001).

**Table 2:** Risk of fracture with or without post-stroke rehabilitation adjusted for covariates.

	Rehabilitation (n = 4192)	Non-rehabilitation (n = 4192)
Subjects with fracture	241	154
Person-years	3840.6	3806.5
Incidence rate*	6.2	4.1
Univariate model		
Crude HR (95% CI)	1.53 (1.25–1.88)	1 (ref.)
p value	<0.001	
Model 1†		
Adjusted HR (95% CI)	1.53 (1.25–1.87)	1 (ref.)
p value	<0.001	
Model 2‡		
Adjusted HR (95% CI)	1.52 (1.23–1.88)	1 (ref.)
p value	<0.001	

* Per 100 person-years, calculated by correcting immortal time.

^†^ Model 1 used a multivariate Cox proportional hazard regression model to adjust for all baseline characteristics listed in Table 1.

^‡^ Model 2 used a stratified Cox proportional hazard regression model by stratifying propensity-score matched groups.

### Anatomic distribution of fractures

The most common fracture sites were vertebra and femoral neck in both groups, accounting for more than 40% of total fractures (Table 3). The rehabilitation group exhibited significantly higher incidence of fractures of vertebral, pelvic, humeral, and femoral neck than non-rehabilitation groups.

**Table 3:** Comparison of fracture sites between post-stroke rehabilitation and non-rehabilitation groups.

Anatomical sites	Rehabilitation (n = 4192)	Non-rehabilitation (n = 4192)	p value
Total fracture events*	241 (5.7%)	154 (3.7%)	<0.001
Skull or facial	5 (0.1%)	7 (0.2%)	0.563
Vertebral	60 (1.4%)	40 (1.0%)	0.044
Rib or sternal	16 (0.4%)	19 (0.5%)	0.611
Pelvic	10 (0.2%)	0 (0%)	0.002
Clavicular or scapular	8 (0.2%)	4 (0.1%)	0.248
Humeral	26 (0.6%)	7 (0.2%)	0.001
Radial or ulnar	14 (0.3%)	20 (0.5%)	0.303
Carpal, metacarpal, or phalanges	14 (0.3%)	9 (0.2%)	0.296
Femoral (neck)	59 (1.4%)	28 (0.7%)	0.001
Femoral (other than neck)	12 (0.3%)	5 (0.1%)	0.089
Patella	4 (0.1%)	3 (0.1%)	0.705
Tibial or fibular	9 (0.2%)	7 (0.2%)	0.617
Ankle	5 (0.1%)	3 (0.1%)	0.479
Tarsal, metatarsal, or phalanges	7 (0.2%)	6 (0.1%)	0.781

A χ2 test was used to compare the differences of fracture rate between the two groups.

*Because there may be more than one fracture site in the same patient, the sum of events of individual fracture sites is not equal to the total fracture events.

### Risk of fractures by sex and age

We further analyzed the association between rehabilitation and fractures stratified for sex and age (Table 4). Among women aged ≥65 years, there was a significantly increased risk of fracture in those undergoing rehabilitation as showed by both multivariate analysis (aHR = 1.62, 95% CI = 1.21–2.17, p = 0.001) and stratified Cox regression model (aHR = 1.82, 95% CI = 1.33–2.50, p < 0.001). No such significantly increased risk was found among the other three subgroups.

**Table 4:** Risk of fractures for patients with and without post-stroke rehabilitation stratified by sex and age.

	Rehabilitation				Non-rehabilitation				Model 1†		Model 2‡	
	n§	Fracture cases	Person-years	Incidence rate*	n§	Fracture cases	Person-years	Incidence rate*	aHR (95% CI)	p value	aHR (95% CI)	p value
**Male**												
**Age 20**–**64**	1039	25	993.2	2.5	1039	30	991.8	3.0	0.85 (0.50–1.45)	0.549	0.87 (0.51–1.49)	0.618
**Age ≥ 65**	1440	64	1313.0	4.9	1440	56	1280.0	4.4	1.12 (0.78–1.61)	0.541	1.24 (0.85–1.81)	0.274
**Female**												
**Age 20**–**64**	476	17	451.7	3.8	476	11	454.9	2.4	1.60 (0.75–3.43)	0.228	1.70 (0.78–3.71)	0.183
**Age ≥ 65**	1172	119	1026.3	11.6	1172	73	1012.6	7.2	1.62 (1.21–2.17)	0.001	1.82 (1.33–2.50)	<0.001

The propensity score matching procedure was performed after initial stratification for sex and age of the total of “11,806 patients”§ included in this study.

* Per 100 person-years, calculated by correcting immortal time.

^†^ Model 1 used a multivariate Cox proportional hazard regression model to adjust for all baseline characteristics listed in Table 1.

^‡^ Model 2 used a stratified Cox proportional hazard regression model stratified for propensity-score matched groups.

^§^ After propensity score matching, a total of 8,384 patients were enrolled. However, the values in the table do not add up to 8,384 patients. This is because in order to keep all paired relationships when stratifying sex and age, we stratified sex and age from the initial total 11,806 patients. After stratification, we separately performed propensity score matching in each stratum. Therefore, there are 2078, 2880, 952 and 2344 patients included in each stratum.

## Discussion

In the present study, we investigated the association of rehabilitation and the risk of fracture following ischemic stroke using a nationwide population database. We observed that patients receiving rehabilitation had a higher incidence of fracture compared with those who did not. This is the first large-scale study to demonstrate an increased fracture risk with rehabilitation after ischemic stroke.

Patients may have fractures after stroke because of fragile bones and falling [36]. Other risk factors besides stroke include older age, female gender, osteoporosis, dementia, depression, immobilization, and inactivity [3, 36, 37]. To account for these factors, we used propensity scores to match differences in baseline characteristics and stroke severity proxies between the two groups. Furthermore, all confounding factors were adjusted for in the multivariate Cox proportional hazard regression models.

Several guidelines suggest that early mobilization and rehabilitation after stroke, including sitting, standing, and walking, yield beneficial effects [38, 39]. Musculoskeletal, cardiovascular, respiratory, and immune systems are negatively affected by bed rest in the acute phase of stroke [40]. Inactive patients are subject to immobility-related complications, including pneumonia, deep vein thrombosis, and pressure sores [19, 41, 42], which may be reduced by early mobilization after acute ischemic stroke [43]. After a stroke, the window of opportunity for brain plasticity and repair may be narrow, making early neurologic rehabilitation optimal [44, 45]. Agility exercise training prevents falls after stroke in older adults by improving postural reflexes, functional balance, and mobility [46]. Muscle and bone strength benefit from rehabilitation, which can help maintain fitness and bone mass, prevent osteoporosis, improve balance, and potentially reduce the risk of falls and fractures [47, 48]. However, although stroke patients benefit from rehabilitation, some do not receive post-stroke rehabilitation. Previous studies have revealed that the rate of rehabilitation usage was approximately 50% in ischemic stroke patients in Taiwan [49, 50]. Chen et al. reported on the possible determinants of rehabilitation for in-patient stroke care [51]. The positive factors for receiving rehabilitation include treatment in a teaching hospital, older age, intracranial hemorrhage, prior surgery, and hemiplegia or hemiparesis; the negative factors include male gender, higher income, higher comorbidity, use of a mechanical ventilator, and participation of a neurologist in patient care.

Even though rehabilitation benefits patients by improving functional status and mobility, we found that it did not protect patients from fractures. Similarly, a meta-analysis study indicated that exercise did not prevent falls after stroke [52]. Simpson et al. found that increasing mobility made patients more vulnerable to falls after stroke. Patients recently discharged from stroke rehabilitation are at a greater risk of falling in their home [53]. Once ambulatory, higher mobility may be associated with incremental odds of falling [54, 55].

A nationwide retrospective cohort study in Sweden reported that 9% of stroke patients had subsequent fractures during a mean follow-up of 2.54 years. The highest fracture risk was during the first year after hospital discharge [56]. A population-based cohort study in Ontario, Canada reported that the 2-year post-stroke fracture rate was 5.7% [57] and another in North Dublin, Ireland reported that 5.4% of patients had fractures in the 2 years after stroke [58]. In this study, the overall fracture rate in the first year after stroke was 4.7%, which is comparable to previous studies.

Patients with rehabilitation had higher incidences of vertebral, pelvic, femoral neck, and humeral fractures than those without rehabilitation; vertebral, femoral neck, and humeral fractures were the most frequent. Vertebral compression fractures are characteristic of osteoporosis and result from low-energy trauma [59]. Femoral neck fractures tend to result from falling to the side or straight down [60], and proximal humeral fractures result from falling obliquely or to the side [61]. Patients receiving post-stroke rehabilitation may have experienced fractures while engaging in mobile activities.

As both age and female gender increase fracture risk [36], we stratified the study groups based on both sex and age. In the rehabilitation group, only older women had a significantly increased fracture risk. Younger women with rehabilitation had a trend toward increased fracture risk but HR was not statistically significant. We did not determine the reason for the difference in fracture rate but suggest that it may have resulted from the high prevalence of osteoporosis in postmenopausal women, which increased vulnerability to low-energy fractures [62]. Apart from osteoporosis, falls and muscle weakness have been associated with fractures in woman [63]. Women may also experience more frequent falls than men. A previous study reported that women had an increased risk of falling because of variation in gait pattern during dual-task activities, which in turn contributed to a greater fracture risk in women compared with men [64]. Fear of falling appeared to be higher in women, and fear of falling in patients after stroke may indicate a person who is at a high risk of falling [65, 66]. According to the Taiwan Ministry of Health and Welfare, the population of single elderly women increased from 19,893 to 25,598 between 2000 and 2012. Single and disabled elderly women may be prone to fractures because of the mobility required for most daily activities. Additional precautions must be taken to prevent fractures in older women receiving post-stroke rehabilitation.

Although we could not determine whether fractures after stroke directly resulted from fall using the administrative database, previous studies have confirmed this hypothesis [5, 36]. Thus, effective interventions for preventing fall should be established to prevent fractures after stroke. Several studies have evaluated strategies to prevent fall, such as physical activity intervention (including balance training, exercise, and strength training) [67–69], environment modification or use of assistive technology [70, 71] and use of different models for stroke care [72]. However, a systemic review concluded that the above mentioned strategies were no more effective than usual care [73]. Moreover, a randomized controlled trial showed that multifactorial fall prevention programs were not effective in reducing this risk in stroke patients [74]. Various drugs increase the risk of falling in the elderly, including sedatives and hypnotics, neuroleptics and antipsychotics, antidepressants, benzodiazepines, and nonsteroidal anti-inflammatory drugs [75]. Medication use was not evaluated in this study, but unnecessary prescription of such drugs should be avoided to reduce the risk of falling after stroke. Therefore, further research is required to identify effective interventions to prevent fall and consequent fractures in stroke patients, especially in those undergoing rehabilitation owing to their higher susceptibility to fractures.

Our study has several advantages. First, it was based on a nationwide population database that includes a representative sample of one million individuals, allowing selection of a sufficient sample size for our research. Second, in contrast to case-control or cross-sectional designs, the retrospective cohort study design generates a higher level of epidemiologic evidence. To eliminate confounding by sociodemographic factors and coexisting medical conditions, we used propensity score matching to balance the groups with and without post-stroke rehabilitation. The use of multivariable as well as stratified Cox proportional hazard models helped control for residual confounding effects. Finally, we calculated the fracture risk in person-years with correction of immortal time to reduce bias [35].

This study has several limitations. First, we could not retrieve lifestyle information; physical, psychiatric, or laboratory data; or marriage and family support status. We also could not determine the actual mechanism of fractures in the absence of additional information to differentiate between intrinsic or extrinsic causes of falls. The actual cause-effect relation between rehabilitation and post-stroke fracture may require further clinical trials. Second, the use of several proxies of stroke severity is not the best way to determine stroke severity. However, we calculated the claims-based SSI score to represent neurologic deficit severity, and this was well matched between the two groups by propensity score matching. A previous study suggested that SSI was an acceptable indicator of severity of ischemic stroke when conducting outcomes research using administrative data [30]. The SSI correlates well with clinical stroke severity measured by the National Institutes of Health Stroke Scale and is significantly associated with the modified Rankin Scale up to 1 year after stroke [31]. Therefore, the difference in stroke severity between the two groups was diminished with adequate matching. However, bias related to confounders might persist in a cohort study. Even if we adequately controlled for known confounders, there might still be bias related to unmeasured or unknown confounders. Finally, the diagnoses in NHI claim records are used for administrative rather than scientific purposes. The anonymized patient data does not allow independent confirmation of diagnoses [76]. Nonetheless, previous studies have reported the high accuracy and validity of ICD-9-CM coding for diagnoses in the database, including stroke and fracture [27, 77]. Hospitals or doctors in Taiwan would be penalized and heavily fined for wrong diagnoses or coding, further establishing the reliability of the diagnoses in the database.

## Conclusions

In summary, we found that patients with ischemic stroke who received post-stroke rehabilitation had a higher risk of fractures compared with patients without rehabilitation, although this risk was increased only in older women. Further investigation is necessary to establish whether preventive measures, such as safety education, environmental modification, and adjustments in rehabilitation programs, would reduce the risk of fractures in patients receiving post-stroke rehabilitation.
